# Rehabilitation interventions in randomized controlled trials for low back pain: proof of statistical significance often is not relevant

**DOI:** 10.1186/s12955-019-1196-8

**Published:** 2019-07-22

**Authors:** Silvia Gianola, Greta Castellini, Davide Corbetta, Lorenzo Moja

**Affiliations:** 1 IRCCS Istituto Ortopedico Galeazzi, Unit of Clinical Epidemiology, Milan, Italy; 2 Department of Biomedical Sciences for Health, University of Milan, Milan, Italy; 3 IRCCS San Raffaele Hospital, Rehabilitation and Functional Recovery Department, Milan, Italy; 4 Vita-Salute San Raffaele University, Physiotherapy Degree Course, Milan, Italy

**Keywords:** Epidemiologic methods, Trials, Randomized clinical minimal clinically important difference, Patient outcome assessment, Data interpretation, Statistical, Sample size

## Abstract

**Background:**

An observed statistically significant difference between two interventions does not necessarily imply that this difference is clinically important for patients and clinicians. We aimed to assess if treatment effects of randomized controlled trials (RCTs) for low back pain (LBP) are statistically significant and clinically relevant, and if RCTs were powered to achieve clinically relevant differences on continuous outcomes.

**Methods:**

We searched for all RCTs included in Cochrane Systematic Reviews focusing on the efficacy of rehabilitation interventions for LBP and published until April 2017. RCTs having sample size calculation and a planned minimal important difference were considered. In the primary analysis, we calculated the proportion of RCTs classified as “statistically significant and clinically relevant”, “statistically significant but not clinically relevant”, “not statistically significant but clinically relevant”, and “not statistically significant and not clinically relevant”. Then, we investigated how many times the mismatch between statistical significance and clinical relevance was due to inadequate power.

**Results:**

From 20 eligible SRs including 101 RCTs, we identified 42 RCTs encompassing 81 intervention comparisons. Overall, 60% (25 RCTs) were statistically significant while only 36% (15 RCTs) were both statistically and clinically significant. Most trials (38%) did not discuss the clinical relevance of treatment effects when results did not reached statistical significance. Among trials with non-statistically significant findings, 60% did not reach the planned sample size, therefore being at risk to not detect an effect that is actually there (type II error).

**Conclusion:**

Only a minority of positive RCT findings was both statistically significant and clinically relevant. Scarce diligence or frank omissions of important tactic elements of RCTs, such as clinical relevance, and power, decrease the reliability of study findings to current practice.

**Electronic supplementary material:**

The online version of this article (10.1186/s12955-019-1196-8) contains supplementary material, which is available to authorized users.

## Background

Randomized controlled trials (RCTs) aim to show differences in an outcome measurement between two or more groups of patients undergoing different interventions [1]. Authors of RCTs usually report findings in term of statistical significance (i.e., with a *p*-value < 0.05 the intervention is more effective than the comparison). However, the p-value indicates the chance of the observed effect, does not consider the magnitude of benefits (or harm) and indeed the clinical relevance. This is defined as the estimate of the smallest treatment effect between groups that people would consider important and is often called minimally important difference (MID) [2].

In physical and rehabilitation medicine and particularity in low back pain (LBP), most of RCTs began as empiric or are based on clinical observation, thus they involved small sample sizes, making more difficult to reach an adequate power to detect a MID between the intervention and control [3–5]. Furthermore, most outcomes are patient-reported and are associated with small clinical changes (e.g., pain reduction from moderate to low). Recognizing small but clinically relevant effects requires clinical trials with large sample sizes. This scenario leads to two problems. First, when a small trial in rehabilitation achieves statistical significance, false positive outcomes may occur. Second, it should not be assumed that trial results which are statistically significant are also clinically relevant [6]. In fact, even if the pre-specified value of success for the primary outcome has been met for the difference in treatment effects (usually a *p*-value of less than 0.05), it does not necessarily imply that the difference matters to patients [7, 8]. For example, a recent re-analysis of data of a published Cochrane review on Multidisciplinary Biopsychosocial Rehabilitation for chronic LBP showed how findings were highly significant but irrelevant in practice. Across studies, pain was reduced by less than one-third of 1 MID unit on a numerical rating scale (0.27 MID units, confidence interval 0.07–0.48) [9]. A MID of 2 points out of 10 is usually considered meaningful [10].

We aimed to assess if treatment effects of RCTs for LBP are both statistically significant and clinically relevant. We also investigated if trials were powered to achieve clinically relevant outcomes assessing the risk of possible false-negative results (i.e., missing an effect that is actually there).

## Methods

This is a cross sectional study, building on a previous published research [4]. We updated the search strategy, adopted the same eligibility criteria and re-run the same selection process. Here, methods are briefly reported.

### Literature search

We used Cochrane Systematic Reviews (SRs) for selecting trials since they are usually considered of high quality, and adopt extensive search strategies. For the identification of Cochrane SRs on LBP, we updated the previous search strategy to April 2017 [4].

### RCTs eligibility criteria

From the eligible Cochrane SRs, we extracted all trials. We considered a trial eligible if it met all the following criteria: (i) was a RCT; (ii) identified a primary outcome and determined the sample size on the basis of the primary outcome; (iii) considered continuous outcomes (e.g., pain, disability) leaving out any binary outcomes (e.g., fall or not fall); (iv) identified a priori planned MID for the primary outcome measure in the sample size calculation; and (v) the language of publication was English or Italian.

### Data collection

We developed an ad hoc data extraction form. For each trial, we collected general information (e.g., country and year of publication) and specific information. Specific information included: the primary continuous outcome (e.g., pain), scales used for the outcome assessment (e.g., numeric pain rating scale), details on measurement scoring (e.g., 0–10 points), planned sample size, planned MID, any bibliographic reference and/or explanation of the rationale for the choice of the MID (e.g. anchor/distribution or other methods), follow-ups, number of randomized patients, number of patients at any follow up. When the time of follow-up analysis was not specified in the sample size calculation, we arbitrarily selected the follow-up time point closest to the end of the intervention.

In addition, we classified the type of intervention as “active treatment” or “inert treatment”, the second used when the expected responses could not be attributed to the investigated interventions (e.g. lack of biological plausibility of an effect). More precisely, we considered the interventions such as manipulation as “active treatment”, while placebo or sham control treatments as “inert treatment”.

Referring to estimates of effect sizes, we noted the mean difference (MD) of the primary outcome and its 95% confidence intervals (CIs), or any other available data to estimate the effect size and its imprecision (e.g. standard errors).

#### Determination of statistical significance

For every comparison between the intervention and control, we dichotomized the statistical significance as ‘achieved’ or ‘not achieved’ according to the pre-specified significance level (i.e., when pre-specified significance level was less than 5%, *p* < 0.05, statistical significance was classified as ‘achieved’).

#### Determination of the clinical relevance

Between-group differences were compared with the planned MID reported in the sample size calculation, determining if the effect size reached clinical relevance. We classified clinical relevance as ‘achieved’ if the point estimate of the MD was equal or greater than the a priori planned MID, and ‘not achieved’ in the other case.

#### Determination of study powered

We defined a study as “powered” if the sample size was equal or greater than the sample size originally planned.

Finally, we screened all RCTs to determine how often authors discussed trial’ findings related to the clinical relevance. We revised all full-text sections and we classified each trial according to the attempt to interpret differences as clinically relevant as “clinical relevance discussed” or “clinical relevance not discussed”. Two reviewers conducted the screening independently and a third author was consulted in case of disagreements.

### Data analysis

We reported data of continuous variables by medians and interquartile range (IQR), and data of categorical variables by frequencies and relative percentages. We computed the number of RCTs falling in each of the following four categories: “*statistically significant and clinically relevant”, “statistically significant but not clinically relevant”, “not statistically significant but clinically relevant”,* and *“not statistically significant and not clinically relevant”*. Whenever multiple arm comparisons were presented, in a primary analysis, we considered the whole trial as *statistically significant* if at least one comparison was statistically significant. In a secondary analysis, we considered each multiple arm comparison as independent.

## Results

### Study selection

**Fig. 1:**
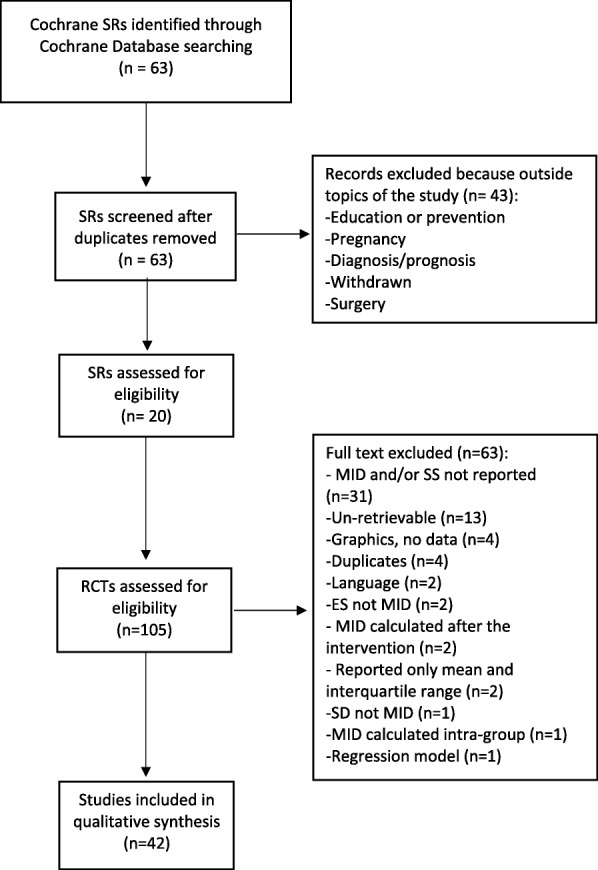
Study selection process. ES = effect size; MID = minimal important difference; RCTs = randimized controlled trials; SD = standard deviation; SRs = systematic reviews; SS = sample size

We identified sixty-three Cochrane SRs. After the selection process, 20 SRs were considered for the identification of eligible trials. Of these reviews, 105 RCTs were eligible from the included Cochrane SRs but only 42 (40%) met the inclusion criteria and were finally included in our study. The study selection process is shown in Fig. 1.

### Trial’s characteristics

The 42 included RCTs were published in 19 journals. Most of these were published in *Spine* (*n* = 13, 31%), in *The British Medical Journal* (*n* = 5, 12%), and in the *Clinical Journal of Pain* (n = 5, 12%). Thirteen countries were designated as publishing countries, of which the most frequent are United States (*n* = 11 RCTs, 26%), United Kingdom (*n* = 9 RCTs, 21%), Norway and the Netherlands (*n* = 4 RCTs, 10%). The publication period runs from 1996 to 2014 (median = 2006; IQR = 2003–2008). Most RCTs reported the funding source (81%). One-fifth of the studies was multi-arm and 29% of trials calculated the sample size based on a composite outcome. One-third of trials (32%) investigated comparisons against an inert intervention. All general characteristics are reported in Table 1.

**Table 1:** General characteristics

	n° of RCT (*n* = 42)	%
N° of countries (*n* = 14)		
United states	11	26
United Kingdom	9	21
Norway	4	10
Netherland	4	10
Brazil	3	7
Australia	3	7
Finland	2	5
Spain	1	2
Sweden	1	2
Switzerland	1	2
Italy	1	2
Thailand	1	2
Taiwan	1	2
N° of journals (*n* = 19)		
Most frequent journals		
Spine	13	31
Clinical Journal of Pain	5	12
British Medical Journal	5	12
Journal of Manipulative and Physiological Therapeutics	3	7
N° of reported funding	34	81
Multi-arm trials	8	19
	n° of comparisons (n = 81)	%
Comparisons		
active treatment versus active treatment	55	68
active treatment versus inert treatment	26	32

### Clinical relevance characteristics

The majority of the included studies (*n* = 37, 88%) reported MID as an absolute value, while the remaining studies reported it as a percentage of improvement over the baseline. Only a half of the included studies (*n* = 20, 48%) referenced the source used to calculated the MID. Eliminating duplicates, 16 different method sources were found and examined. Of these, 6 were anchor-based, one distribution-based, one based on consensus (e.g. expert panel), three cited other articles, three were not clearly described and information was not found in two studies.

### Is the effect always clinically relevant?

Table 2 shows the main findings for statically significant and clinically relevant results. We found that almost a half of trials (*n* = 16, 40%) were “not statistically significant and not clinically relevant” and more than a half (*n* = 25, 59%) were statistically significant. Out of these 25, 15 trials (36% of all included trials) were both “statistically significant and clinically relevant” and 10 trials, (24% of all included trials) were “statistically significant but not clinically relevant”. One trial was classified as “not statistically significant but clinically relevant”.

**Table 2:** Statistically significance and clinically relevance on continuous outcomes of LBP. Δ is the MID. Negative values means improvement (for example, greater pain reduction in the treatment vs. control group)

Scenario	N° of trials (%) (total = 42)	N° of comparisons (%) (total = 81)
A) statistically significant and clinically relevant	15 (36%)	20 (25%)
B) statistically significant but not clinically relevant	10 (24%)	22 (27%)
C) not statistically significant but clinically relevant	1 (2%)	1 (1%)
D) not statistically significant and not clinically relevant	16 (38%)	38 (47%)

Considering all comparisons of multiple arm trials (*n* = 81) the four scenarios were similar to those reported in Table 2. However, among statistical positive findings (scenario A and B, *n* = 42 out of 81), a consistent part of the comparisons were against an inert treatment (40%) as compared to active head-to-head comparisons (60%) (Additional file 1: Table S1).

### Is the clinical relevance always discussed?

Eighteen out of 42 trials (43%) did not report or discuss the clinical relevance of their results, even when clinical relevance was demonstrated (5%). Table 3 lists most frequent types of omissions and embellishments characterising reporting of results when clinical relevance was not reached and considered. Full omission for the primary outcome was the main strategy (11 trials).

**Table 3:** Types of omissions and embellishments in reporting RCT findings when clinical relevance was not reached and considered (*n* = 42)

Clinical Relevance discussion	Strategy for specific reporting	No. (%)
Clinical relevance discussed		24 (57)
Clinical relevance not discussed		18 (43)
not reached		16 (39)
	Full omission for the primary outcome	7 (44) ^a^
	Full omission for all primary outcomes used in the sample size calculation^b^	4 (25) ^a^
	Clinical relevance discussed only as within-group improvements	4 (25) ^a^
	Clinical relevance discussed at follow-ups not declared in the sample size calculation	1(6) ^a^
reached		2 (5) ^a^

^a^ The Total refers to 16 trials that not discussed the clinical relevance

^b^Composite outcomes

### Were non-statistically significant interventions powered?

Four studies did not report the number obtained in the sample size calculation. For the other 38 studies, the median of the sample sizes planned a priori was 125 subjects, while the median of the actual enrolled sample sizes was 133 subjects. Nevertheless, 14 trials out of 38 (37%) reached the planned sample size, while remaining were low-powered. Sixteen out of 38 trials (42%) do not achieve the statistical significance but less than half of these (*n* = 6, 40%) have an adequate and powered sample size (Table 4, scenarios C and D).

**Table 4:** Statistical and clinical effects according to planned a priori sample size achievement

Scenario per n° of trials (total = 38^a^)	N° of powered trials (%)	N° of unpowered trials (%)
A) statistically significant and clinically relevant (*n* = 14)	7 (50)	7 (50)
B) statistically significant but not clinically relevant (*n* = 8)	1 (12)	7 (87)
C) not statistically significant but clinically relevant (*n* = 1)	0	1 (100)
D) not statistically significant and not clinically relevant (*n* = 15)	6 (40)	9 (60)
Totals	14 (37)	24 (63)

^a^The total number of trials is 38 because four studies did not report the patients number obtained from the sample size calculation (1 study belongs to scenario A, 2 belong to scenario B and 1 study belongs to scenario D)

## Discussion

Based on the RCTs included in our retrospective cohort, we found a poor reporting of rehabilitation interventions for LBP in terms of validity and clinical relevance. In fact, only a half of the trials reported the source reference of the adopted planned MID as measure of validity of the clinical relevance and, among studies achieving statistically significant results (*n* = 25/42), only 60% (*n* = 15/25) achieved the planned clinical relevance. This means that 1 out of 2 studies reaching a statistically significant difference favoring a treatment has results that cannot be truly relevant for stakeholders, clinicians and patients. This result could also be overestimate because the 29% of trials reported the sample size based on composite outcomes at risk of falling in type I error. Less than a half of RCTs in our sample (43%) did not discussed their findings from a clinical perspective, mainly by omitting information, particularly when the clinical relevance was not reached. Moreover, we found that in trials reaching statistically significant and clinically relevant results a consistent part of multi-arm comparisons were against inert treatments. All these findings support the hypothesis that the efficacy of rehabilitation interventions for LBP tend to be overestimated, or potentially underestimated if we considered that 63% trials with not statistically significant and not clinically relevant results did not have an adequate powered sample size.

Our results are coherent with the literature where the reporting of results in terms of clinical relevance is sparsely used across trials [1]. We confirm the preliminary results published by Van Tulder et al. focused on exercise therapy for chronic LBP reporting that less than half of studies (39%) with positive conclusions shown clinically important differences [11]. A general poor reporting of clinical relevance is also present across pharmacological interventions with a discussion of results in clinical terms ranging from 24 to 46% of the samples [12–15].

When results of trials do not achieve a statistically significant and/or a clinically relevant difference among treatments, authors tends to discuss and shape the impression of their results for readers. In scientific writing, this is called “to spin” the scientific report [16]. In our sample of RCTs, the most frequently adopted strategy to spin the report was the under-reporting of the clinical relevance of founded statistically significant results. One possible reason at the basis of this phenomenon can be found in the publication process of biomedical research that tends to favor the publication of positive results [17]. To some extent, similarly for statistical significance, it can happens that reports of RCTs with clinically relevant results are published more often than those with not-clinically relevant results [18, 19].

Clinical relevance can influence not only the statistically significant results but also the non-statistically ones. In fact, the aim to detect a MID between the intervention and control group determines the power of study in the sample size calculation. A study conducted in 2008 in the field of physical medicine and rehabilitation reported that, of the 82 articles reviewed, 57% reported sample size calculation and 13% of them without sufficient information about the parameters required for a priori calculation [3]. In a more recent published study on low back pain rehabilitation we denounced a low frequency of trials reporting all elements needed for sample size calculation [4]. Anyway, also taking into account all trials with all the elements for sample size calculation, we now found a very low percentage of powered trials that clinically interpreted their findings on the light of the planned clinical relevance. This issue encompass other healthcare professions than the physical rehabilitation in the evidence based care: a large proportion of the existing trials are poorly designed and underpowered [20]. The potential weakness in small-size “negative” clinical trials was already reported and pointed out fifty year ago [6].

### Strength and limitations

The major strength of this study is to assess the clinical relevance of results assuming the MID declared in the sample size of each study and not a standardized MID as already previously investigated [11]. However, some limitations are present. The sample of trials only included non-pharmacological LBP interventions and our findings may not be extended to other trials published on different interventions (e.g., pharmacological interventions). Moreover, we did not assess the risk of bias in each trial that could have been correlated the quality of study to the interpretation of results.

### Implication for research

In a planning phase of a clinical trial, scientific ethics committees should be more rigid on the sample size definition requiring its “a priori” calculation and its complete reporting. Ethics committees should mandatory require researchers to provide the preliminary data or a referenced study assessing the MID of the outcome used for the determination of the sample size calculation. This is expected to happen also for pilot studies, even if exposed to unexplored knowledge. The size of a pilot study should be calculated in relation to the desired level of confidence for the SD and the chosen power and significance level of analysis in the main study. At high level of confidence, a pilot study of at least *n* = 50 is advisable in many circumstances [21]. The same process should be followed during the editorial assessment of a scientific report before its publication. An accurate replication of the sample size should be done, prior to the approval of experimental study. Thus, we called for powered trials in order to avoid approximated, wrong and unfounded assumptions ensuring a sufficiently large sample size to draw meaningful conclusions, even if studies with larger sample sizes are more onerous in terms of both time and money.

We call for more adherence to reporting of planned sample size including the clinical relevance with the clinical interpretation of the effects. Without the complete information, the reader is unable to fully interpret the results of a study [1]. On one hand, authors have to report all elements used for sample size calculation, including the clinical relevance. Furthermore, they have the duty to coherently interpret observed effects on the light of this threshold. Otherwise, sample size calculation does not make any sense. On the other hand, editors and reviewers have to enforce authors to provide sufficient details about clinical relevance and sample size calculation (sample planned, randomized and reached) for the primary outcome.

This would prevent an unusable treatment for its non-interpretable effects and leading to promote treatments clinically relevant. Then, the actual guidelines for the reporting of patient-reported outcome in RCTs, endorsed through the initiatives of the CONSORT Statement [22, 23] promote the discussion (item 22) of a minimal important change in the interpretation of patient-reported outcome results. Actually, clinical relevance is not explicitly contemplated in the planning: the current item 7 only describe “how sample size was planned”. The reporting of patient-reported outcome in RCTs must consider to expand the item regarding the sample size definition introducing a dedicate section for the declared a priori clinical relevance and reporting of its validity (e.g., by citation of references). This approach can avoid too positive results improving the assessment of the imprecision of quality of evidence and standardizing the process. If findings are statistically significant but not clinically important, the quality of evidence in the meta-analyses and guidelines will potentially change the conclusions [24].

We also suggest researchers to select a single primary endpoint for formal statistical inference otherwise involving several outcomes in conventional significance testing can seriously inflate the overall type I error rate [15].

### Implication for clinical practice

A very low proportion of trials research (2.6%) reflects the priorities of patients and clinicians showing an important mismatch between wishes of patients and evaluations of researchers [25]. Treatments efficacy should be useful in terms of clinical relevance. This would allow a better informing patients strategy on the possible benefits and harms of the intervention, as well as their size, costs, and inconveniences of the intervention for a tailored therapy. The shared decision-making approach should encompass the patient’s preferences and values into the discussion in a perspective evidence based health-care [26]. A treatment leading to non-relevant results for patients is often an unsuccessful treatment, resulting in frustration, discontinuation of therapies and waste of resources. An approach focused on the achievement of a clinically relevant effect of a treatment will increase awareness of condition and the participation of each patient in the managing of their benefit-harm trade-off tailored, limiting the burden of physical rehabilitation conditions for obsolete or harmful or discontinued treatments.

## Conclusions

Authors’ conclusions are usually too positive. The clinical relevance and the power of study are not yet fully considered as a valid measures reported in the sample size, and in the interpretation of findings of RCTs in LBP rehabilitation. If authors of trials reported adequately the a priori sample size and commented their results in term of clinical relevance, the threshold for efficacy could identify the real meaningful effect. Otherwise the common justification of “not enough power to detect a significant efficacy of the intervention” is always justifiable for negative studies.

## Additional file

Additional file 1: **Table S1**. Positive and negative results in the intervention comparisons (*n* = 81). (DOCX 15 kb)

## Data Availability

All data generated or analysed during this study are included in this published article [and its Additional files].

## Abbreviations

CI: Confidence Interval
CONSORT: Consolidated Standards of Reporting Trials
ES: Effect Size
LBP: Low Back Pain
MID: Minimal Important difference
RCT: Randomized Controlled Trial
SD: Standard Deviation
SR: Systematic Reviews
SS: sample size
