# Supplementary material for: Rehabilitation interventions in randomized controlled trials for low back pain: proof of statistical significance often is not relevant
Source: Health Qual Life Outcomes. 2019 Jul 22;17:127. doi: 10.1186/s12955-019-1196-8 (PMC6647152; doi:10.1186/s12955-019-1196-8)
Supplement: Supplementary file 1 — Table S1. Positive and negative results in the intervention comparisons (n = 81). (DOCX 15 kb) [file 12955_2019_1196_MOESM1_ESM.docx]

**Table S1**. Positive and negative results in the intervention comparisons (n=81).

| **All comparisons (n=81)** | **Statistical positive findings**  **N=42** | | **Statistical negative findings**  **N=39** | |
| --- | --- | --- | --- | --- |
| **Active versus active comparisons**  **n ( %)** | **25 (31)** | | **30 (37)** | |
|  | scenario A | 8 (10) | scenario C | 1(1) |
|  | scenario B | 17 (21) | scenario D | 29 (36) |
| **Active versus inert comparisons**  **n ( %)** | **17 (21)** | | **9 (11)** | |
|  | scenario A | 12 (15) | scenario C | 0 (0) |
|  | scenario B | 5 (6) | scenario D | 9 (11) |

**Legend**. A “statistically significant and clinically relevant”; B “statistically significant but not clinically relevant”; C “not statistically significant but clinically relevant”; D “not statistically significant and not clinically relevant”
